# Tomato Rootstocks Mediate Plant-Water Relations and Leaf Nutrient Profiles of a Common Scion Under Suboptimal Soil Temperatures

**DOI:** 10.3389/fpls.2020.618488

**Published:** 2021-01-21

**Authors:** Steven T. Bristow, Leonardo H. Hernandez-Espinoza, Maria-Sole Bonarota, Felipe H. Barrios-Masias

**Affiliations:** Department of Agriculture, Veterinary, and Rangeland Sciences, University of Nevada, Reno, Reno, NV, United States

**Keywords:** root hydraulic conductivity, stomatal conductance, root anatomy, mineral nutrition, grafting, *Solanum lycopresicum* L

## Abstract

Environments with short growing seasons and variable climates can have soil temperatures that are suboptimal for chilling-sensitive crops. These conditions can adversely affect root growth and physiological performance thus impairing water and nutrient uptake. Four greenhouse trials and a field study were conducted to investigate if rootstocks can enhance tomato performance under suboptimal soil temperatures (SST). In a controlled greenhouse environment, we exposed four commercial rootstocks (Estamino, Maxifort, RST-04-106-T, and Supernatural) grafted with a common scion (cv. BHN-589) to optimal (mean: 24°C) and SST (mean: 13.5°C) and compared their performance with the non-grafted BHN-589 cultivar. Several root and shoot physiological traits were evaluated: root hydraulic conductivity and conductance, root anatomy, leaf gas exchange, leaf δ^13^C, shoot C and N, and biomass. Under field conditions, the same five phenotypes were evaluated for canopy growth, normalized difference vegetation index (NDVI), leaf nutrients, biomass, and yield. Under SST, root hydraulic conductivity (*Lp*) and conductance (*K*_R_), stomatal conductance (*g*_s_), and plant biomass decreased. Hydrostatic *Lp* decreased more than osmotic *Lp* (*Lp*^∗^_hyd_: 39–65%; *Lp*^∗^_os_: 14–40%) and some of the reduced conductivity was explained by the increased cortex area of primary roots observed under SST (67–140%). Under optimal soil temperatures, all rootstocks conferred higher *g*_s_ than the non-grafted cultivar, but only two rootstocks maintained higher *g*_s_ under SST. All phenotypes showed greater reductions in shoot biomass than root biomass resulting in greater (∼20%) root-to-shoot ratios. In the field, most grafted phenotypes increased early canopy cover, NDVI, shoot biomass, and fruit yield. Greenhouse results showed that *Lp*^∗^_os_ may be less affected by SST than *Lp*^∗^_hyd_ and that reductions in *Lp* may be offset by enhanced root-to-shoot ratios. We show that some commercial rootstocks possess traits that maintained better rates of stomatal conductance and shoot N content, which can contribute toward better plant establishment and improved performance under SST.

## Introduction

Tomato (*Solanum lycopersicum* L.) is a highly nutritious crop produced globally and under a wide array of abiotic and biotic stresses. For thermophilic crops such as tomato, production is especially challenged in regions where low temperatures are a significant environmental factor determining the cropping season (Schwarz et al., 2010). For tomatoes, temperatures below 20°C are considered suboptimal (Van Ploeg and Heuvelink, 2005). Suboptimal soil temperatures (SST) can affect root establishment, water and nutrient uptake, and overall plant performance under field and greenhouse conditions (Abdelhafeez et al., 1971; Hurewitz and Janes, 1983; Tindall et al., 1990; Venema et al., 1999; Bloom et al., 2004; Onwuka and Mang, 2018). The regions prone to SST, especially in the spring, are those with high annual or diurnal temperature variations and are typically arid such as Mediterranean, desert, and high-elevation environments (Stoller and Wax, 1973; Dai et al., 1999; Sanders and Markhart, 2000). In the spring, air temperatures fluctuate close to the optimal conditions for shoot function, but root physiology is still challenged by SST (Walter et al., 2009). This occurs when daily fluctuations in soil temperatures lag air temperatures, which can be up to 6 h at just 10 cm depths (Zheng et al., 1993). Thus, root performance under SST becomes critical (Schwarz et al., 2010), especially early in the growing season, and the use of rootstocks that are less susceptible to SST could provide desirable traits to overcome some of the limitations for cold-sensitive vegetables in regions where soil warming is slow or farmers consider earlier plantings to avoid extreme heat during the summer.

Root systems of chill-tolerant genotypes better maintain plant-water relations and nutrient uptake under suboptimal rootzone temperatures (SRT; e.g., in hydroponic systems) as shown for *Zea mays* L. (maize), *Cucumis sativus* L. (cucumber), and tomatoes (Ahn et al., 1999; Aroca et al., 2003; Ntatsi et al., 2014). When plants simultaneously encounter optimal air temperatures and SRT, root systems may be unable to meet shoot water demands as movement of water through the root can be inhibited. However, chill-tolerant *Cucurbita ficifolia* Bouché (figleaf gourd) showed improved water uptake capacity [i.e., root hydraulic conductivity (*Lp*)] under SRT when compared to chill-sensitive cucumber (Lee et al., 2005a,b). The improved *Lp* of figleaf gourd under SRT was related to slower suberization of the endodermis and sustained aquaporin permeability (i.e., less gating) (Lee et al., 2005a,b). In maize, a chill-tolerant cultivar demonstrated faster recovery of the hydrostatic hydraulic conductivity (*Lp*_hyd_), better maintenance of osmotic hydraulic conductivity (*Lp*_os_), and sustained transpiration under SRT (Aroca et al., 2001). As SST may last several weeks after planting, the ability of roots to adjust their water uptake capacity becomes critical to prevent prolonged plant stress and delayed establishment. Studies have shown that chill tolerance to short-term exposures (i.e., hours) to SRT requires a fast reduction of stomatal conductance (*g*_s_) to prevent shoot desiccation (e.g., tomato: Bloom et al., 2004), while longer exposures would require acclimation of the root *Lp* along with *g*_s_ (e.g., spinach: Fennell and Markhart, 1997, maize: Aroca et al., 2001). Faster acclimation of root *Lp* could result in higher rates of *g*_s_ and carbon assimilation under SRT leading to earlier plant establishment (Ahn et al., 1999). When cucumber was grafted onto figleaf gourd, it improved g_s_ and photosynthetic rates (*P*_n_) under SRT (Ahn et al., 1999). In tomato, the chill-tolerant *Solanum habrochaites* reduced *g*_s_ faster than the chill-sensitive cultivar after SRT exposure (Bloom et al., 2004). Wild-type tomato relatives have shown their potential to improve tolerance to SRT (Venema et al., 2008; Ntatsi et al., 2017) and are being used in breeding for commercial rootstocks (King et al., 2010; Suchoff et al., 2018). However, limited information is available regarding the hydraulic capacity [e.g., root hydraulic conductance (*K*_R_) and *Lp*] of commercial rootstocks and their capacity to confer increased tolerance to SST for tomatoes.

Root anatomical and morphological characteristics are also important factors influencing root performance under abiotic stress, and root traits such as small root diameters and increased root length density have been associated with improved nutrient and water uptake and increased plant productivity under drought (Eissenstat, 1992; Comas et al., 2013). In tomato, low temperatures result in shorter and thicker roots, and rootstocks that increase root length can improve plant performance (Ntatsi et al., 2014). Under SRT, thinner lateral roots and greater branching was reported in a chill-tolerant maize genotype (Ciamporová and Dekánková, 1998). As SRT lowers root physiology and elongation, root maturation could occur closer to the root tip (e.g., earlier Casparian band) and increases in the root-to-shoot ratio are usually observed (Schwarz et al., 2010). In wheat, smaller increases in the root-to-shoot ratios under chilling exposure was found in winter varieties compared to spring varieties (Equiza et al., 1997, 2001), suggesting a higher functionality of the root, per unit of mass, in winter varieties. Under long-term exposure to SRT, grafting of a sensitive tomato cultivar onto *S. habrochaites* increased the root-to-shoot ratio and promoted root and shoot growth (Venema et al., 2008; Ntatsi et al., 2014, 2017). Plant nutrient content is also compromised because of the reduced root growth, root surface area, changes in membrane fluidity, and increased nutrient efflux back to the soil (Abbas, 2012). In tomato, both macro- and micronutrient uptake have been shown to be diminished in soil temperatures below 25°C (Tindall et al., 1990). SST can reduce N mineralization rates (Zak et al., 1999) and SRT can reduce N uptake (Toselli et al., 1999). Total P uptake was shown to decrease with lower soil temperatures regardless of the solubility of the P fertilizer applied (Case et al., 1964), likely due to reduced root growth limiting the capacity for nutrient interception (Mackay and Barber, 1984). Nutrient deficiencies can decrease root hydraulic conductivity and affect the movement of water through the soil-plant-atmosphere continuum (Clarkson et al., 2000). Relative to *S. lycopersicum, S. habrochaites* showed improved ammonium uptake after short-term exposure to SRT (Smart and Bloom, 1993; Bloom et al., 1998). Suitable rootstocks can improve root branching and surface area which are traits associated with increased nutrient and water uptake (Huang et al., 2016) and have been shown to confer tolerance to SST to a scion (Ahn et al., 1999). Rootstocks have been shown to alter nutrient uptake in tomatoes by either increasing or decreasing the uptake of specific nutrients (Leonardi and Giuffrida, 2006; Savvas et al., 2011; Schwarz et al., 2013) and can increase the utilization efficiency of nutrients such as N in tomato (Djidonou et al., 2013) and other cucurbits such as watermelon (Nawaz et al., 2017).

This study investigated the capacity of various commercial rootstocks to improve performance of a commercial cultivar under several weeks of continuous SST exposure in both greenhouse and field conditions. Our objectives were to: (1) evaluate several root traits that have a direct impact on plant-water relations; (2) assess the capacity of the rootstocks to improve tolerance to SST based on several root and shoot traits; and (3) understand how different root systems may affect the nutrient profile and yield of a common scion. Rootstocks were sourced from different developers (www.vegetablegrafting.org; Kleinhenz, 2017) and several trials were conducted in a greenhouse where only roots were exposed to SST to compare the performance of the grafted phenotypes to a non-grafted cultivar. A field experiment was subsequently conducted in hoop houses to determine if performance in the greenhouse correlated with increased performance in the field.

## Materials and Methods

### Plant Material and Experimental Setup

Four greenhouse trials were conducted at the Valley Road Greenhouse Complex, and one hoop house experiment at the Main Station Field Lab both at the University of Nevada, Reno. Greenhouse trials were conducted from February 2018 through July 2019. The field experiment was conducted from May through September 2018. For all experiments, the tomato cultivar BHN-589 (BHN) was used as both a scion and as the non-grafted cultivar. We chose to use a non-grafted “control” because adopters of this technology would likely consider the benefit of a rootstock against the own-rooted cultivar. Research shows that even though grafting may influence plant performance, the overall impact may be minimal (Djidonou et al., 2013; Suchoff et al., 2018; Asins et al., 2017). For instance, Lang et al. (2020) found no differences between non-grafted and self-grafted plants concerning biomass, fruit yield and quality under field conditions. Fullana-Pericàs et al. (2020) also showed no difference in photosynthetic parameters between reciprocal-grafted and non-grafted plants under non-stress conditions. BHN-589 was grafted onto the commercial tomato rootstocks Estamino (EST), Maxifort (MAX), RST-04-106-T (RST), and Supernatural (SUP) (all combinations and the non-grafted cultivar are referred to as “phenotypes”). Greenhouse trials altered soil temperature between treatments; air temperature was kept at ∼26°C. The treatments included two soil temperatures: suboptimal soil temperature (∼13.5°C) and optimal soil temperature (OST; ∼24°C). For three greenhouse trials, plants were prepared *in situ* using a splice grafting method. For the field and the second round of the greenhouse trials, plants were provided by Plug Connection (Vista, CA, United States), which used a similar grafting method as applied *in situ*.

### Greenhouse Trials

Plants prepared *in situ* were germinated in a 1:2 mixture of seed starter mix (Miracle-Gro, OH, United States) and 30 grit sand (Quikrete, GA, United States). Scion and rootstocks were germinated 2 weeks prior to the non-grafted plants. After ∼21 days from germination, plants were grafted and placed into a GEN1000 growth chamber (Conviron, Winnipeg, MB, Canada) to heal for up to 2 weeks. Seedlings were fertilized twice weekly with 40 ml of 4-12-4 fertilizer (N-P-K) diluted in 15 L of water (Miracle-Gro, OH, United States). After healing, plants were transplanted into square pots (7 cm width × 23 cm height; total volume of 960 ml; Stuewe and Sons, Inc., OR, United States) filled with a 1.5 cm base layer of fritted clay to facilitate drainage and 20 cm of sand.

After transplanting, plants under the SST treatment were placed into modified horizontal refrigerators (HBB-95-HC 95″, AVANTCO, PA, United States) where only the pot (i.e., soil and roots) were exposed to the cold temperature. The refrigerators maintained the soil to an average temperature of ∼13.5°C (Recorded minimum and maximum temperatures: 11 and 18°C). The control temperature plants were placed on benches adjacent to the refrigerators and average soil temperatures were 24°C (Recorded minimum and maximum temperatures: 21 and 32°C). Soil temperatures were measured at two points, 1 cm below the soil surface and 1 cm above the fritted clay, using HOBO U23-003 soil temperature sensors (Onset Computer Corp., MA, United States). The greenhouse air temperature was set to 28 and 21°C (day and night). Average day length was 14 h and supplemental lighting was used when days were shorter. Relative humidity varied between 30 and 40%. Plants were watered daily to field capacity with a fertilizer solution of 3.5 g of 20-20-20 per liter of water (JR Peters, Inc., PA, United States). Greenhouse plants were setup in a split-plot design with temperature treatment as main plot (trial one contained 1 bench and 1 refrigerator, trial 2, 3, and 4 contained 2 benches and 2 refrigerators).

### Hoop House Trial

Six-week-old plants were transplanted into four hoop houses when soil temperatures were still below optimal. Each hoop house contained four beds with five plots per bed and four plants per plot (i.e., each bed represented a replicate of all phenotypes; 20 plots total per hoop house). Plants were spaced 40 cm apart with a 1.2 m spacing between the centers of the beds. All data was collected from the two central beds in each hoop house (10 plots) and predominantly from the two central plants in each plot. In each hoop house, one 5TE probe per bed was installed at 20 cm soil depth (Meter Group, WA, United States) to record volumetric soil water content and temperature. Air temperature and relative humidity were recorded 1.5 m above the soil surface in two of the hoop houses using HOBO U23 Pro v2 (Onset Computer Corp., MA, United States). All data loggers recorded every 30 min. Plants were drip irrigated once per week for several hours depending on soil moisture and weather conditions to maintain volumetric water content at approximately 22%. Plants were fertilized via drip tape twice with 30 kg ha^–1^ (60 units total) of N at 33 and 71 days after planting (DAP) with Phytamin^®^ Fish Plus (4.5-2-1; California Organic Fertilizers, CA, United States).

Soil temperature at 4 and 8 cm depths were estimated using the equation obtained from Koorevaar et al. (1983, pg. 196–197) with calculations corrected by 20 cm soil depth and air temperatures. Temperatures are provided for the 1000 h measurement as air temperature and light intensity should be within the optimal range for plant function. At the 20 cm depth, soil temperatures were only below 18°C at 15, 16, and 17 DAP. At both the 4 and 8 cm depths, the estimated soil temperature was consistently lower than 18°C until 24 DAP. Until 24 DAP, the average air temperature was ∼26°C (min: 14, max: 33). Mid-late season soil temperature was consistently below 18°C starting at 55 DAP for 8 cm, 72 DAP for 4 cm, and 102 DAP for 20 cm. After 55 DAP, average air temperature was 30°C (min: 22°C, max: 34°C). For the entire season, average air temperature was 30°C (min: 14°C, max: 36°C).

### Root Hydraulic Conductivity and Conductance

Root hydrostatic and osmotic hydraulic conductivity (*Lp*^∗^_hyd_ and *Lp*^∗^_os_), as well as hydrostatic and osmotic conductance (*K*_R–hyd_ and *K*_R–os_), were evaluated in intact root systems of plants from all four greenhouse experiments. Plants were harvested for *Lp* measurements within 4 weeks after exposure to the temperature treatments and separate plants were used for hydrostatic or osmotic *Lp* protocols. For the calculation of root *Lp*, measurements were normalized using fresh root biomass in place of root surface area and denoted with “^∗^,” which is consistent with Hernandez-Espinoza and Barrios-Masias (2020; and references therein). Prior to 800 h, plants were moved from the greenhouse to the laboratory and fully watered at least one hour prior to measurements. Plants were detopped by cutting below the graft union, and under water to prevent cavitation. For *Lp*^∗^_os_, a piece of Tygon^®^ tubbing was fitted to the stem and sap was collected every 30 min from the tubing with a pipette and weighed to estimate volume. Five collections were taken from each plant, and a linear regression fitted to estimate the slope of the relationship between time and volume of sap exuded. The collected sap was frozen until the osmolality was measured using a vapor pressure osmometer (Vapro 5600, ELITechGroup Biomedical Systems, Logan, UT, United States). For *Lp*^∗^_hyd_ measurements, entire root systems were carefully washed to not break any roots and then placed in a plastic container filled with water inside a specialized pressure chamber that was pressurized by a Scholander-type pressure chamber (600 EXP; PMS Instruments, Albany, OR, United States). Measurements were taken at 0.05, 0.1, 0.15, 0.2, and 0.25 MPa. Roots acclimated for 10 min at each pressure, and then a dry pre-weighed gauze pad was placed directly over the protruding stem for 10 min to absorb the exuded water. The gauze pad was weighed, and the volume of water estimated. Calculations were performed as outlined by Barrios-Masias et al. (2015).

### Root Anatomy

Roots from greenhouse trials 2, 3, and 4 used only in the *Lp*^∗^_os_ measurements were preserved in 50% ethanol and gradually increased to 90% over 4 weeks. Samples were kept at 4°C until processed. Primary roots were free-hand sectioned at 1 and 3 cm from the root apex and staining was conducted according to Knipfer et al. (2020) using Toluidine Blue-O (Acros Organics, NJ, United States). Images of cross sections were taken using a Leica DFC-295 digital camera for bright field images as well as with a LEICA thunder imager with a DAPI filter for fluorescence images (Leica, Hesse, Germany). The polygon selection tool in ImageJ (Fiji software) was used to measure the xylem vessel, stele and cortex areas (Schindelin et al., 2012). Three representative xylem vessels were selected, and their areas were averaged per cross section only when lignified xylem was present. Stele area was measured as the area within, and not including the endodermis. If the endodermis was not discernable, it was assumed to be the first ring of cells outside the stele. Cortex area was measured as the area between the outside of the endodermis and inside of the exodermis. Cortex cell layers were counted between the endodermis and exodermis in three directions and averaged. Average cortical cell area was calculated for each cross-section by dividing the cortex radius by the number of cortical layers to estimate the average cortical cell diameter.

### Leaf Gas Exchange

Stomatal conductance (*g*_s_) was measured using a Decagon SC1 leaf porometer (Meter Group, WA, United States) on a mature leaflet adjacent to the terminal leaflet on the third or fourth leaf from the top of the plant. Measurements were conducted between 1200 and 1400 h on plants in experiments 1, 2, and 4. Plants were measured on consecutive dates for 3–5 days the week prior to root hydraulic measurements.

Photosynthetic rate (*P*_n_) was measured using a field portable open flow infrared gas analyzer (model 6400, LI-COR Inc., NE, United States). Measurements were taken between 1000 and 1200 h on plants in the fourth experiment for four consecutive days the week prior to root hydraulic measurements. The photosynthetic photon flux density was set to 2000 μmol m^–2^ s^–1^, the reference CO_2_ concentration was set at 400 μmol-CO_2_ mol^–1^, and the block temperature was set at 24°C.

### Carbon, Nitrogen, and δ^13^C

Samples from trials 3 and 4 were analyzed for δ^13^C (180 total samples) utilizing a Micromass Isoprime stable isotope ratio mass spectrometer (Isotopx, NM, United States) at the UNR Stable Isotope Lab. Shoot samples from greenhouse trials 2, 3, and 4 (100 total samples) were analyzed using a CN928 series macro-combustion instrument for C and N (LECO, MI, United States) at the USDA-ARS-Great Basins Rangelands Research’s soils laboratory.

### Plant Biomass

For greenhouse trials, roots were gently cleaned of soil particles, patted dry, and then fresh weight was recorded after root hydraulic measurements were completed. Fresh roots and shoots were placed in an oven at 60°C and dry weight was recorded after 48 h. Field biomass evaluations were conducted at 126 DAP. The two central plants in each plot were cut at the base of the stem and separated into shoots and fruits. All fresh biomass was weighed in the field and then subsamples for each plot were dried at 60°C. Percent of subsample dry weights were used to calculate total dry weights.

### Canopy Cover and NDVI

Early season soil canopy cover and normalized difference vegetation index (NDVI) values were measured on the two central beds and two central plants per plot in each hoop house. Measurements were quantified using an Agricultural Digital Camera (TETRACAM Inc., CA, United States). Pictures were pre-processed in PixelWrench2 (TETRACAM Inc., CA, United States) and then imported to R software for analysis (R Core Team, 2019). Extracting pixels with NDVI values greater than zero was successful for separating canopy from soil. Extracted pixels divided by total pixels was used to calculate the percent cover which was converted into area cover using a formula derived from the field of view calculator in PixelWrench2. For each picture, pixels with NDVI values greater than the 80th percentile were subset to separate mature leaves from stems and older leaves. Subsequently, 2000 random pixels were selected and averaged for comparison among phenotypes. Canopy cover measurements do not correct for overlapping canopy.

### Leaf Nutrient Content

Eight leaflets per field plot were harvested three times during the season (26, 62, and 126 DAP). Harvests approximately correspond to plant establishment, anthesis/fruit set, and post peak production. Leaflets were harvested without petioles from the second or third fully expanded and mature leaf. Leaflets were dried at 60°C for 48 h and 0.3–0.5 g of homogenized material was digested using the protocol from Handbook of Reference Methods for Plant Analysis (Miller, 1998; pp 53–62). After digestion, plant nutrient contents (B, Ca, Cu, Fe, Mg, Mn, P, K, Na, and Zn) were quantified using a microwave plasma-atomic emission spectrometer (MP-AES) (Agilent Technologies, CA, United States) at the USDA-ARS Great Basin Rangelands Research’s soils laboratory. Leaflet samples for C and N from 26 and 62 DAP were unfortunately lost, while samples from 126 DAP were analyzed as described above.

### Statistical Analysis

The effect of “temperature,” “phenotype,” and their interaction (fixed effects) on the response variables was investigated using linear mixed-effect model (lme4 package) in R 3.6.1 (R Core Team, 2019). Random effects were selected using Akaike information criterion, AIC, (ANOVA function, base R) and in nesting order: “trial,” “block,” and “main plot” for the greenhouse experiment. In addition, repeated measures analysis (e.g., *g*_s_, *P*_n_, cortex and stele area) included “pot” (subject) and “day” or “tip distance” (within-subject) as random effects. For all field experiment models, “phenotype” was the fixed effect and “hoop house” was the random effect. For all models, the alpha for main effects was set 0.05. *Post hoc* multiple-comparison procedures were conducted using the multcomp function (emmeans package) using unrestricted least significant difference (LSD) test (Saville, 2015). A stricter alpha of 0.01 was set per null comparison for greenhouse data. Field data was analyzed similarly, except due to the smaller sample size and fewer possible comparisons (10), an alpha of 0.05 was used.

Leaf nutrient data was analyzed using a linear discriminant analysis to identify which nutrients and at which time points best differentiated between the phenotypes (lda function, MASS package). Prior to the analysis, data was transformed into standard normal variables (mean: 0, standard deviation: 1) to receive standardized discriminant unit coefficients from the model (McCune et al., 2002).

## Results

### Greenhouse

#### Root Hydraulics

**FIGURE 1:**
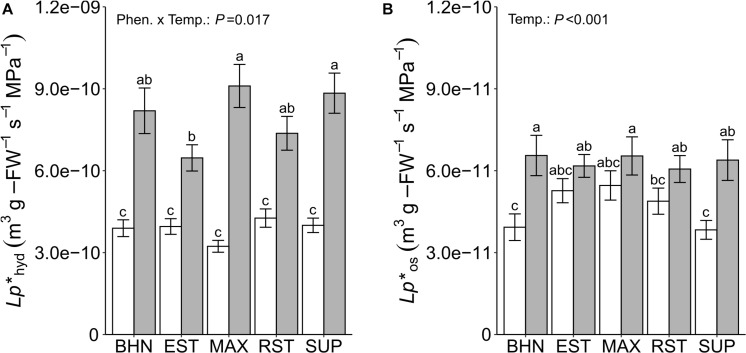
Root hydrostatic (Lp*_hyd_; **A**) and osmotic (Lp*_os_; **B**) hydraulic conductivity on whole-root systems of four grafted rootstocks (Estamino, Maxifort, RST-04-106-T, and Supernatural) and one cultivar (BHN-589) grown under optimal (gray) and suboptimal (white) soil temperatures for 4 weeks. All rootstocks were grafted with BHN-589. Values are mean ± standard error (**A:** *n* = 25–31; **B:** *n* = 23–31). Means followed by different letters are statistically different at *P* < 0.01.

Exposure to SST reduced *Lp*^∗^_hyd_ for all phenotypes although an interaction effect between temperature treatment and phenotype was observed (Figure 1A). However, SST only reduced *Lp*^∗^_os_ for two phenotypes compared to their OST counterparts (Figure 1B). The reductions in *Lp*^∗^_hyd_ ranged from 39 to 65% for EST and MAX, respectively. The only differences in *Lp*^∗^_hyd_ among phenotypes was EST having a lower *Lp*^∗^_hyd_ than MAX and SUP under OST. *Lp*^∗^_os_ was significantly affected by temperature, but to a lesser extent than *Lp*^∗^_hyd_; only BHN and SUP showed reductions in *Lp*^∗^_os_ between soil temperature treatments by ∼40%. There were no differences between phenotypes within either temperature treatment. However, EST and MAX under SST maintained a similar *Lp*^∗^_os_ as BHN in OST.

**FIGURE 2:**
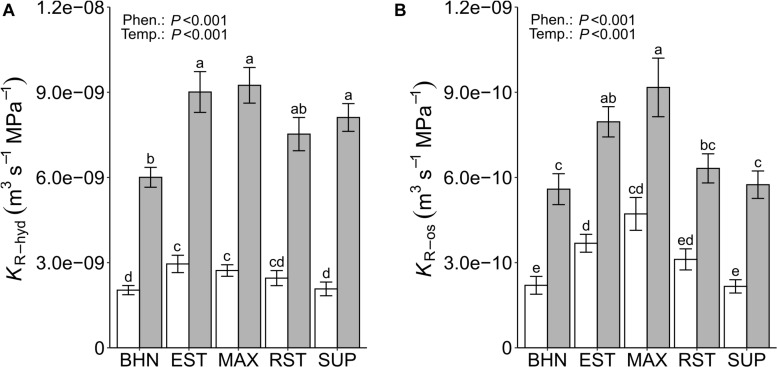
Root hydrostatic (*K*_R__–hyd_; **A**) and osmotic (*K*_R__–os_; **B**) hydraulic conductance on whole-root systems of four grafted rootstocks (Estamino, Maxifort, RST-04-106-T, and Supernatural) and one cultivar (BHN-589) grown under optimal (gray) and suboptimal (white) soil temperatures for 4 weeks. All rootstocks were grafted with BHN-589. Values are mean ± standard error (**A:** *n* = 28–34; **B:** *n* = 24–29). Means followed by different letters are statistically different at *P* < 0.01.

Similar to *Lp*^∗^_hyd,_ *K*_R–hyd_ was lower under SST than under OST for all phenotypes (Figure 2A). The *K*_R–hyd_ under SST decreased between 66 and 74% (BHN and SUP, respectively), compared to their OST counterparts. In the OST treatment, the *K*_R–hyd_ of EST, MAX, and SUP was at least 26% higher than BHN. Whereas under SST, EST and MAX were at least 24% greater than both BHN and SUP. Unlike *Lp*^∗^_os_, the *K*_R–os_ under SST decreased for all five phenotypes between 49 and 62% for MAX and SUP, respectively (Figure 2B). Under OST, EST, and MAX had at least 30% greater *K*_R–os_ than BHN. Whereas under SST, the *K*_R–os_ of EST and MAX were 40 and 53% greater than BHN, respectively. MAX also maintained a similar *K*_R–os_ under SST as BHN under OST, while RST and SUP did not differ from BHN in either soil temperature treatment.

#### Root Anatomy

Exposure to SST increased the thickness of primary roots as indicated by the increased cortex and stele areas (Table 1). Plants exposed to SST had between 67 and 140% (MAX and BHN, respectively) larger cortex areas than their OST counterparts. Stele area increased between 50 and 129% for MAX and SUP, respectively. The only difference in cortex area between phenotypes was MAX having 40% more area than RST under OST. Stele area showed more differences between phenotypes. Under OST, MAX had at least 36% greater stele area than EST and RST. Whereas under SST, BHN, MAX, and SUP had at least 46% greater area than EST.

**TABLE 1:** Cortex area, stele area, cortical cell layers, and cortical cell area of four grafted rootstocks (Estamino, Maxifort, RST-04-106-T, and Supernatural) and one cultivar (BHN-589) exposed to optimal and suboptimal soil temperatures for 4 weeks.

**Phenotype**	**Soil temperature**	**Cortex area (mm^2^)**	**Stele area (mm^2^)**	**Cortex**	
				**Layers**	**Cell area (μ m^2^)**
BHN-589	Suboptimal	0.63 ± 0.07a	0.069 ± 0.007a	7.38 ± 0.3*ab*	2.85 ± 0.1*abc*
	Optimal	0.26 ± 0.03*cd*	0.031 ± 0.004*cde*	5.75 ± 0.5d	2.07 ± 0.2d
Estamino	Suboptimal	0.51 ± 0.04*ab*	0.042 ± 0.002*bc*	7.20 ± 0.2*ab*	2.47 ± 0.2*abcd*
	Optimal	0.28 ± 0.04*cd*	0.026 ± 0.003e	5.88 ± 0.4*cd*	2.07 ± 0.3d
Maxifort	Suboptimal	0.69 ± 0.06a	0.061 ± 0.004a	7.63 ± 0.3a	2.92 ± 0.1*ab*
	Optimal	0.41 ± 0.04*bc*	0.041 ± 0.003*bcd*	6.50 ± 0.4*bcd*	2.44 ± 0.2*abcd*
RST-04-106T	Suboptimal	0.50 ± 0.05*ab*	0.052 ± 0.006*ab*	7.25 ± 0.3*ab*	2.44 ± 0.3*abcd*
	Optimal	0.24 ± 0.02d	0.024 ± 0.003e	5.50 ± 0.3d	2.04 ± 0.2d
Supernatural	Suboptimal	0.60 ± 0.08*ab*	0.064 ± 0.008a	6.88 ± 0.4*abc*	3.10 ± 0.2a
	Optimal	0.31 ± 0.03*cd*	0.028 ± 0.004*ed*	5.88 ± 0.1*cd*	2.25 ± 0.2*cd*
Phenotype		*P* = 0.003	*P* < 0.001	*P* = 0.079	*P* = 0.099
Treatment		*P* < 0.001	*P* < 0.001	*P* < 0.001	*P* < 0.001
Phenotype × Temperature		*P* = 0.628	*P* = 0.106	*P* = 0.586	*P* = 0.708

*Values are means ± standard error and letters that follow indicate statistical differences, within the column, at *P* < 0.01. Phenotype, temperature, and interaction effect *P*-values for each parameter are shown at bottom of table.*

The increased cortex area under SST was explained by an increase in both the number of cortex layers and an increase in the average cortical cell area (Table 1). The number of cortex cell layers increased under SST for all phenotypes except SUP. Increases in cell layers ranged from about 1 for MAX to about 2 layers for BHN, EST, and RST. Average cortical cell area only increased for BHN and SUP under SST by about 38%. Neither the number of cortex layers nor the average cortical cell size differed between phenotypes under either temperature treatment. Although stele area increased under SST, no changes in total xylem area nor xylem count were observed (data not shown).

Root maturation and development as determined by exodermis suberization and xylem lignification was not affected by SST (Table 2). However, greater presence of the Casparian band closer to the root tip (1 cm) was observed under SST than under OST (52% vs. 35%). For both temperature treatments, all roots at 3 cm from the tip showed 100% presence of the indicators of root development.

**TABLE 2:** Presence of Casparian band, suberized exodermis, and lignified xylem in roots exposed to optimal and suboptimal soil temperatures for 4 weeks and sectioned at 1 and 3 cm from the apex.

**Tip distance**	**Soil temperature**	**Percent presence**		
		**Casparian band**	**Suberized exodermis**	**Lignified xylem**
1cm	Suboptimal	52%	76%	33%
	Optimal	35%	75%	35%
3cm	Suboptimal	100%	100%	100%
	Optimal	100%	100%	100%

*Phenotypes were grouped together to better explore the influence of suboptimal soil temperatures on root anatomy.*

#### Gas Exchange

**FIGURE 3:**
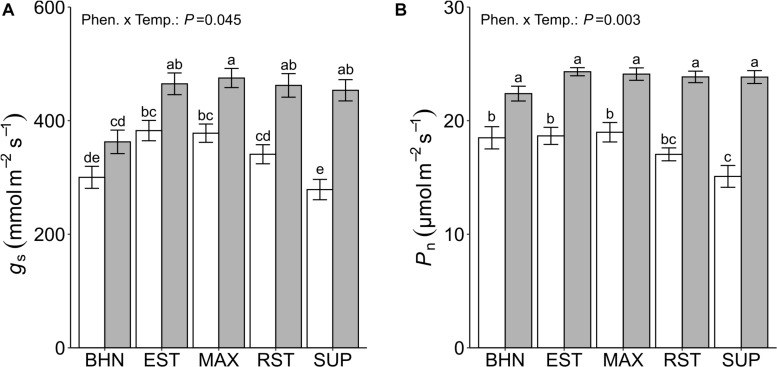
Stomatal conductance (*g*_s_; **A**), and photosynthetic rate (*P*_n_; **B**) of four grafted rootstocks (Estamino, Maxifort, RST-04-106-T, and Supernatural) and one cultivar (BHN-589) grown under optimal (gray) and suboptimal (white) soil temperatures for 4 weeks. All rootstocks were grafted with BHN-589. Values are mean ± standard error (**A:** *n* = 14 and 48 total measurements; **B:** *n* = 6 and 24 total measurements) Means followed by different letters are statistically different at *P* < 0.01.

Overall, exposure to SST reduced *g*_s_ and *P*_n_ although an interaction effect between temperature treatment and phenotype was observed. Stomatal conductance decreased for MAX, RST, and SUP under SST (Figure 3A). These reductions in *g*_s_ ranged from 20% for MAX to 39% for SUP. Under OST, the *g*_s_ rates of all grafted phenotypes were approximately 20% greater than BHN. While under SST, *g*_s_ for EST and MAX was at least 26% greater than BHN. Except for SUP, the *g*_s_ of all phenotypes under SST was similar to the rates of BHN under OST.

The *P*_n_ was lower for all phenotypes under SST than under OST (Figure 3B). The differences between treatments ranged from 17 to 37% for BHN and SUP, respectively. Phenotypes did not have different *P*_n_ under OST, while under SST only SUP was at least 19% lower than BHN, EST, and MAX.

The leaf δ^13^C increased under SST between 6.8 and 8.7% for EST and SUP, respectively (Table 3). No differences in δ^13^C among phenotypes were observed under OST. Under SST, the only difference was SUP having a 3.5% greater (less negative) δ^13^C than MAX. No other differences were observed.

**TABLE 3:** Leaf δ^13^C, shoot percent carbon, shoot percent nitrogen, shoot biomass, and root biomass of four grafted rootstocks (Estamino, Maxifort, RST-04-106-T, and Supernatural) and one cultivar (BHN-589) exposed to optimal and suboptimal soil temperatures for 4 weeks.

**Phenotype**	**Soil temperature**	**δ ^13^C**	**Shoot carbon (%)**	**Shoot nitrogen (%)**	**Biomass (g plant^–1^)**	
					**Shoot**	**Root**
BHN-589	Suboptimal	−28.6 ± 0.5*ab*	40.2 ± 0.1a	4.6 ± 0.3c	1.8 ± 0.1*cb*	0.47 ± 0.02*fe*
	Optimal	−30.7 ± 0.6c	38.7 ± 0.3*dc*	5.9 ± 0.3*ab*	2.9 ± 0.1a	0.64 ± 0.03*cd*
Estamino	Suboptimal	−28.9 ± 0.4*ab*	39.4 ± 0.3*abc*	5.4 ± 0.3*ab*	1.9 ± 0.1b	0.60 ± 0.03*cd*
	Optimal	−31.0 ± 0.5c	38.3 ± 0.3d	6.2 ± 0.2*ab*	3.3 ± 0.1a	0.84 ± 0.03a
Maxifort	Suboptimal	−29.2 ± 0.4b	38.9 ± 0.3*bcd*	5.4 ± 0.3*ab*	1.8 ± 0.1*cb*	0.59 ± 0.03d
	Optimal	−31.3 ± 0.4c	38.2 ± 0.3d	6.3 ± 0.3a	3.1 ± 0.1a	0.82 ± 0.03*ab*
RST-04-106T	Suboptimal	−28.7 ± 0.3*ab*	39.3 ± 0.3*abc*	5.3 ± 0.3*bc*	1.8 ± 0.1*cb*	0.54 ± 0.03*de*
	Optimal	−31.2 ± 0.5c	38.2 ± 0.4d	6.0 ± 0.2*ab*	3.1 ± 0.1a	0.80 ± 0.04*ab*
Supernatural	Suboptimal	−28.2 ± 0.5a	39.7 ± 0.3*ab*	4.6 ± 0.3c	1.6 ± 0.1c	0.44 ± 0.03f
	Optimal	−30.9 ± 0.4c	38.2 ± 0.3d	5.9 ± 0.3*ab*	2.9 ± 0.1a	0.70 ± 0.03*bc*
Phenotype		*P* = 0.021	*P* = 0.011	*P* = 0.006	*P* = 0.005	*P* < 0.001
Treatment		*P* < 0.001	*P* < 0.001	*P* = 0.001	*P* < 0.001	*P* < 0.001
Phenotype × Temperature		*P* = 0.572	*P* = 0.582	*P* = 0.325	*P* = 0.724	*P* = 0.479

*Values are means ± standard error and letters that follow indicate statistical differences, within the column, at *P* < 0.01. Phenotype, temperature, and interaction effect *P*-values for each parameter are shown at bottom of table.*

#### Biomass

**FIGURE 4:**
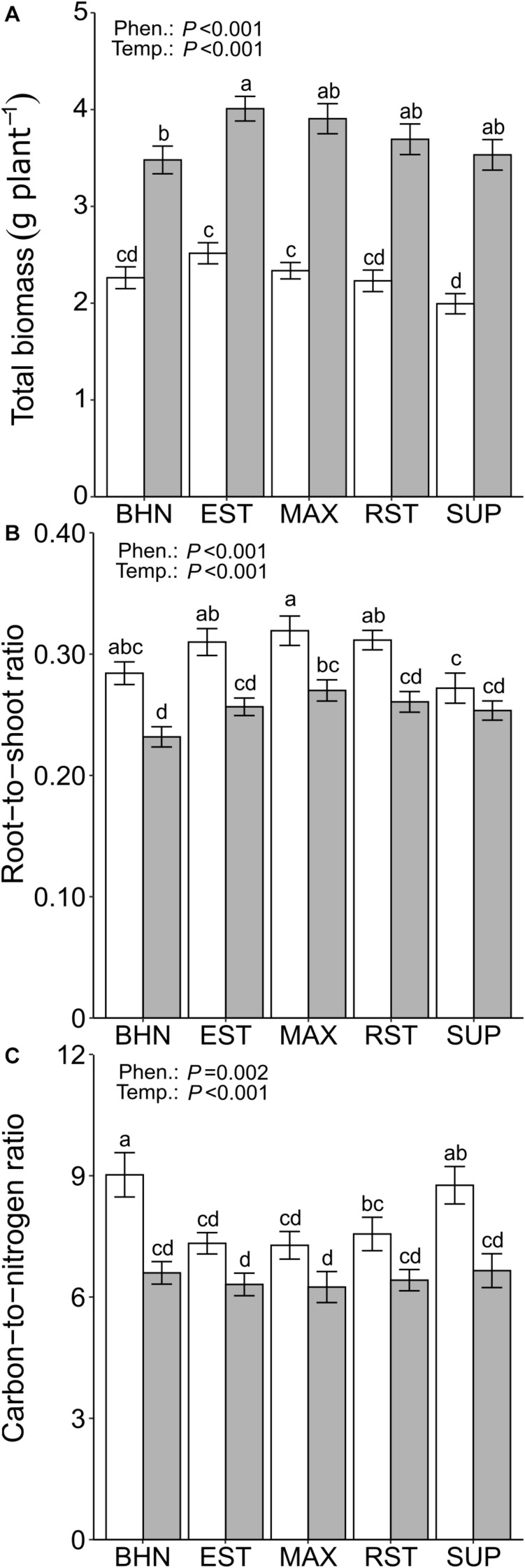
Total biomass (root + shoot; **A**), root-to-shoot ratio **(B)**, and C-to-N ratio **(C)** of four grafted rootstocks (Estamino, Maxifort, RST-04-106-T, and Supernatural) and one cultivar (BHN-589) grown under optimal (gray) and suboptimal (white) soil temperatures for 4 weeks. All rootstocks were grafted with BHN-589. Values are mean ± standard error (**A,B**: *n* = 45–50; C: *n* = 10). Means followed by different letters are statistically different at *P* < 0.01.

Suboptimal soil temperatures reduced plant biomass for all phenotypes (Figure 4A). BHN had the smallest reduction in total biomass of 35% while SUP had the largest reduction of 44%. Under OST, EST had 32% greater total biomass than BHN and no other differences were observed. Under SST, BHN’s total biomass was similar to all phenotypes while EST and MAX had 25 and 17% more than SUP, respectively. Shoot biomass did not differ between phenotypes under OST (Table 3). Under SST, EST had 21% greater shoot biomass than SUP. In contrast, root biomass showed more differences between phenotypes for both soil temperature treatments. Under OST, EST, and MAX had at least 29% more root biomass than BHN and at least 17% more than SUP. Under SST, EST, and MAX had at least 24% more than BHN and at least 33% more than SUP (Table 3). EST, MAX, and RST all had similar root biomass under SST as BHN had under OST.

Suboptimal soil temperatures resulted in greater reductions in shoot biomass than in root biomass (42% vs. 30%), leading to an increase in the root-to-shoot ratio for all phenotypes (except SUP) relative to OST (Figure 4B). The root-to-shoot ratios increased between 18 and 23% for MAX and BHN, respectively. Under OST, MAX had a 17% greater root-to-shoot ratio than BHN. Under SST none of the phenotypes differed from BHN, but EST, MAX, and RST were at least 14% greater than SUP.

#### Carbon and Nitrogen

Under SST, the C concentration in the shoot increased compared to the OST counterparts for all phenotypes except MAX (Table 3). Those increases in C ranged between 2.8 and 3.8% for EST and BHN, respectively. Under OST, the C concentration did not differ between any of the phenotypes, whereas under SST, MAX had 3% less C than BHN; no other differences were observed. EST, MAX, and RST, under SST, had similar C concentrations to BHN under OST.

Suboptimal soil temperatures only reduced the shoot N concentrations in BHN and SUP and by at least 22% compared to their OST counterparts (Table 3). Under OST, the N concentration did not differ among phenotypes. Under SST, MAX, and EST had around 18% more N than both BHN and SUP, while RST did not differ from any other phenotype. Similar to C concentrations, EST, MAX, and RST, under SST, maintained similar concentrations of N to BHN under OST.

Suboptimal soil temperatures led to a 37 and 32% increase in the C-to-N ratio for BHN and SUP, respectively (Figure 4C). Under OST, no difference in C-to-N ratio was observed between phenotypes, whereas under SST, EST, MAX, and RST were all at least 16% lower than BHN and 14% lower than SUP. EST, MAX, and RST under SST also maintained similar C-to-N ratio as BHN under OST.

### Hoop House

#### Soil Canopy Cover, NDVI, Biomass and Fruit Yield

**FIGURE 5:**
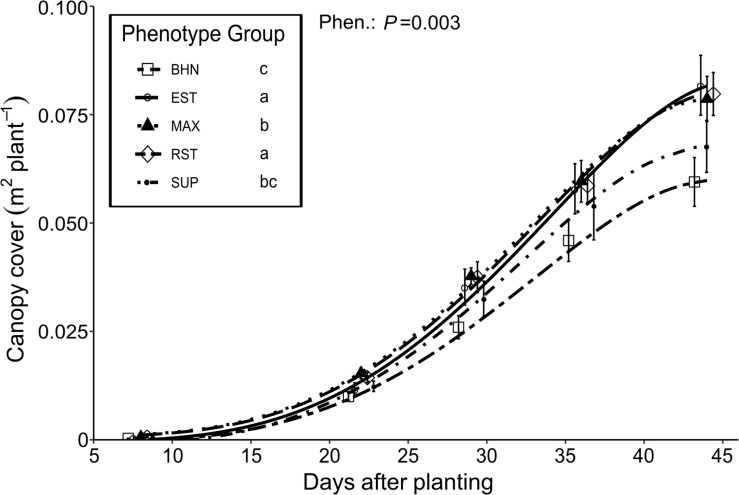
Changes in canopy cover of four grafted rootstocks (Estamino, Maxifort, RST-04-106-T, and Supernatural) and one cultivar (BHN-589) grown under field conditions. All rootstocks were grafted with BHN-589. All canopies were measured at 8, 22, 29, 36, and 44 days after planting (*x*-values staggered for visual clarity). Canopy cover values were inverse-negative-log transformed prior to slope comparisons. Data presented is untransformed. Points are mean ± standard error (*n* = 7–8). Legend information followed by different letters are mean-slopes statistically different at *P* < 0.05.

Soil canopy cover remained similar and less than 10 cm^2^ plant^–1^ between 0 and 8 DAP (Figure 5). From 8 to 44 DAP, the average daily increases in soil canopy cover ranged from 16 to 23 cm^2^ day^–1^ for BHN and EST, respectively. The slopes of the increase in soil canopy cover for EST, MAX, and RST were 37, 19, and 34% steeper than BHN, respectively. The growth of SUP was slower than EST and RST but did not differ from BHN or MAX. Between 8 and 22 DAP, the average daily increase in soil canopy cover ranged between 7 and 11 cm^2^ day^–1^ for BHN and MAX, respectively. From 22 to 29 DAP, the average daily increases in soil canopy cover was at least three times faster than before and ranged from 23 to 34 cm^2^ day^–1^ for BHN and EST, respectively. Between 29 and 36 DAP, soil canopy cover increased for BHN to 28 cm^2^ day^–1^ but remained similar for the other phenotypes (∼30 cm^2^ day^–1^). After 36 DAP, the average daily increase in soil canopy cover began to decrease for BHN, MAX, RST, and SUP while EST appeared to maintain the same growth rate of ∼30 cm^2^ day^–1^.

**FIGURE 6:**
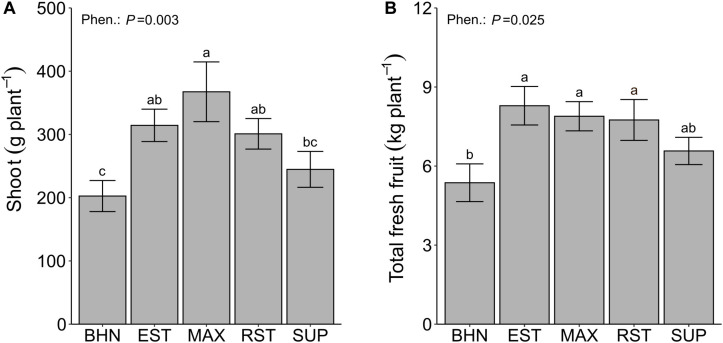
Shoot dry biomass **(A)**, and total fresh fruit **(B)** of four grafted rootstocks (Estamino, Maxifort, RST-04-106-T, and Supernatural) and one cultivar (BHN-589) grown under field conditions. All rootstocks were grafted with BHN-589. Values are mean ± standard error (**A,B**: *n* = 8). Means followed by different letters are statistically different at *P* < 0.05.

Normalized difference vegetation index values consistently increased for all phenotypes from 8 to 44 DAP (Table 4). Over that time, BHN started with the highest NDVI value but ended with the lowest value. BHN ranged from 0.61 to 0.74 for a total increase of 0.13. In contrast, EST and SUP both started with the lowest values and ended being similar to the other grafted phenotypes with a total increase in NDVI of ∼0.18. RST and MAX both started similar to BHN but ended being greater with a total increase of ∼0.16. Overall, all grafted phenotypes had at least 4% higher NDVI values than BHN by 44 DAP. EST, MAX, and RST had higher shoot biomass and fresh fruit than BHN while SUP was similar (Figures 6A,B). EST, MAX, and RST had 55, 81, and 49% more shoot biomass and 54, 47, and 44% more fruit than BHN, respectively.

**TABLE 4:** Normalized Difference Vegetation Index (NDVI) of four grafted rootstocks (Estamino, Maxifort, RST-04-106-T, and Supernatural) and one cultivar (BHN-589) grown under field conditions and measured at 8, 22, 29, and 44 days after planting (DAP).

**Phenotype**	**NDVI (DAP)**			
	**8**	**22**	**29**	**44**
BHN-589	0.610 ± 0.005a	0.667 ± 0.008c	0.710 ± 0.003f	0.736 ± 0.008d
Estamino	0.586 ± 0.003b	0.675 ± 0.008c	0.717 ± 0.011*ef*	0.764 ± 0.006g
Maxifort	0.604 ± 0.006a	0.682 ± 0.007c	0.729 ± 0.004*de*	0.765 ± 0.005g
RST-04-106T	0.595 ± 0.008*ab*	0.680 ± 0.005c	0.722 ± 0.003*def*	0.769 ± 0.004g
Supernatural	0.579 ± 0.005b	0.671 ± 0.003c	0.720 ± 0.005*ef*	0.761 ± 0.008g

*Data is mean ± standard error (*n* = 7–8). Legend information followed by different letters are means statistically different at *P* < 0.01. Phenotype *P* = 0.002.*

#### Nutrients

**FIGURE 7:**
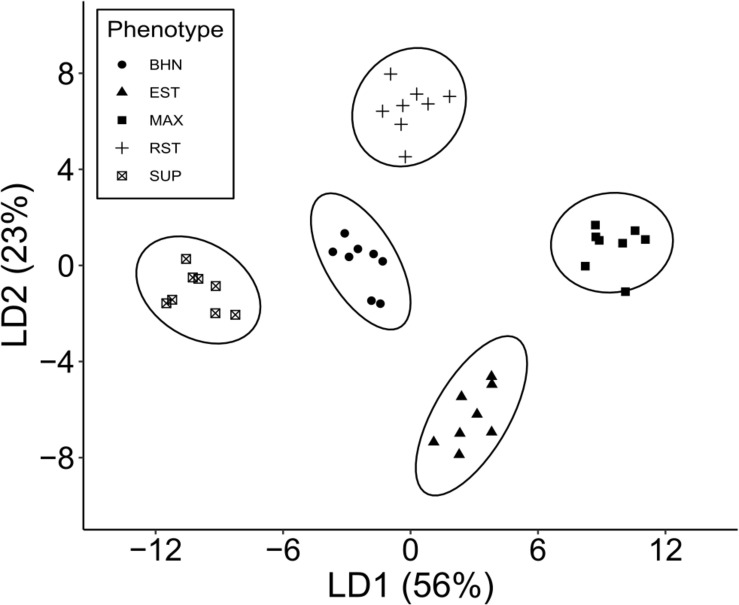
Linear discriminant analysis based on ten nutrients (B, Ca, Cu, Fe, K, Mg, Mn, Na, P, Zn) at 3 times points (26, 62, 126 DAP) and C and N at 126 DAP for four grafted phenotypes (Estamino, Maxifort, RST-04-106-T, and Supernatural) and one cultivar (BHN-589) (*n* = 8). Total variance explained by the first two discriminant functions are presented in parentheses.

Linear discriminant 1 (LD1) accounted for 56% of the separation between phenotypes and its most important features were predominantly macronutrients at the earliest sampling (Figure 7). P, K, Ca, and Mg content at 26 DAP were four of the five most important features as well as Mn at 62 DAP (see Supplementary Material 1 for standardized discriminant unit coefficients). Mean values for P, K, Ca, and Mn were typically higher in the grafted phenotypes than in the non-grafted cultivar except for Mg, which was highest in SUP and BHN (Supplementary Material 2). The next five most important features were also mainly macronutrients, but at 126 DAP (K, N, and Mg) as well as one micronutrient at 26 (Na) and 62 (Zn) DAP. Along the LD1 axis, SUP is well separated from BHN, EST, and RST, which are also separated from MAX.

Linear discriminant 2 (LD2) accounted for 23% of the variation and its most important features were micronutrients at various time points (Figure 7). Na, K, and Cu at 62 DAP were three of the five most important features as well as Cu at 126 and B at 26 DAP (Supplementary Material 2). The next five most important features were also micronutrients from either 26 (Zn, Na) or 62 (Fe, B, Mn) DAP. The mean concentrations of micronutrients appeared to vary by phenotype and nutrient with no consistent pattern. Along the LD2 axis, RST is separated from BHN, MAX, and SUP, which are also separated from EST.

Linear discriminant 3 and 4 (LD3 and LD4) account for 17 and 4% of the variation and were both defined by macro- and micronutrients at various time points. Of the five most important features in LD3, the macronutrients were Mg at 62 DAP and K and N at 126 DAP, while the micronutrients were Mn and B at 62 DAP. Along the LD3 axis, BHN is well separated from all the grafted phenotypes but no other separation was apparent (axis not shown). LD4 alone did not offer good separation between any phenotypes (axis not shown).

## Discussion

Although rootstock vigor is usually referred as the cause of improved cultivar performance, this study shows specific traits associated with grafting that enhanced tomato performance under SST. Primarily, root traits associated with improved water relations, nutrient uptake, and biomass accumulation supported growth of the common scion. Overall, all phenotypes were sensitive to SST, and changes in physiological and morphological traits such as lower water uptake capacity of roots (i.e., root *Lp* and *K*_R_), increased root thickness (i.e., cortex and stele area), and decreased leaf gas exchange were observed. Yet, under SST some of the commercial rootstocks maintained higher *K*_R_ and *g*_s_ and a better shoot nutrient profile of the common scion, which were likely favored by smaller reductions in root biomass than the non-grafted cultivar. Similarly, under field conditions, grafted phenotypes had greater canopy growth and NDVI values early in the season, which are indicative of better root growth and establishment, and resulted in greater shoot and fruit biomass by the end of the season. Strategies for managing crops under SST are still insufficient for production in locations with short growing seasons and variable conditions, but our study shows that some commercial rootstocks possess traits important for improved crop establishment that are associated with enhanced water and nutrient uptake under SST.

Exposure to SST reduced water uptake, and this has been related to increases in water viscosity and physiological changes such as membrane permeability or reduced energy metabolism (Bloom et al., 2004). Our study shows that SST reduced water movement through the roots as indicated by ∼50% reductions in *Lp*^∗^_hyd_ and subtle decreases in *Lp*^∗^_os_. Those reductions are partly explained by observed changes in root morphology and anatomy such as increased cortex area (i.e., increased cortex cell size and number of cell layers) and presence of a Casparian band closer to the root tip, which contributed to increase the resistance along the apoplastic pathway (Steudle and Peterson, 1998; Rieger and Litvin, 1999). Although not quantified in this study, a further explanation to the reduced *Lp*^∗^_hyd_ may include a reduction in root branching associated with prolonged exposure to SST (Kaspar and Bland, 1992; Koevoets et al., 2016), which would likely reduce the root length density. Moreover, reductions in *K*_R–hyd_ under SST were more pronounced than the decreases in *Lp* and at ∼70% of OST. The greater reduction of *K*_R–hyd_ likely occurred due to a combined effect of lower *Lp*^∗^_hyd_ and less root biomass. This suggests that early root growth becomes important for plant establishment under SST in order to increase the total capacity for water uptake, as observed in MAX and EST, which maintained greater *K*_R–hyd_ than the non-grafted cultivar.

While hydrostatic-driven flow was similarly affected in all phenotypes, osmotic-driven flow was only reduced in the lower performing phenotypes under SST (BHN and SUP). This indicates a differential response in water uptake capacity through the cell-to-cell pathway, which can help maintain water status under abiotic stress (Steudle and Peterson, 1998; Barrios-Masias et al., 2019). Studies on cucurbits and maize under SST showed that improved root hydraulics was associated with sustained aquaporin permeability or increased aquaporin content (Aroca et al., 2001; Lee et al., 2005a,b). Although aquaporin activity was not measured in this study, it is likely that it played a role in lowering the resistance to water movement through the cell-to-cell pathway for some of the rootstocks. In addition, the capacity to acclimate to suboptimal temperatures can improve osmoregulation and chilling tolerance (e.g., watermelon; Lu et al., 2020). For instance, the non-grafted cultivar showed a larger increase in the sap osmotic potential than MAX under SST (data not shown), but the *Lp*^∗^_os_ in the former still decreased relative to its OST counterpart, suggesting that resistance to water movement increased despite osmotic adjustment. MAX showed a smaller increase in the sap osmotic potential but still maintained a similar *Lp*^∗^_os_ under both temperature treatments. Chilling sensitivity results in part from an impaired capacity of the root to supply water to a transpiring shoot (Bloom et al., 2004; Koevoets et al., 2016), but this study suggests that root acclimation and the additive effect of sustained *Lp*^∗^_os_ with only slight reductions in root biomass resulted in better capacity to meet transpirational demands (i.e., *K*_R_) and favor plant water relations in early growth stages.

Gas exchange measurements showed SST affected *g*_s_ more than *P*_n_ in some of the phenotypes, suggesting that uptake and transport of water played an important role in shoot performance. Although the responses of *g*_s_ and *P*_n_ are considered to be closely correlated (Matthews et al., 2017), *g*_s_ can be more sensitive to stress (e.g., drought; Barrios-Masias et al., 2018). In tomato and grapevine, rootstocks proportionally increased *g*_s_ more than *P*_n_ (Koundouras et al., 2008; Fullana-Pericàs et al., 2018), consistent with our observation that under OST *g*_s_ increased more than *P*_n_ for all grafted phenotypes. However, under SST, MAX and EST had higher *g*_s_ than other phenotypes, and EST, MAX, and RST showed no differences in stem water potential compared to their OST counterparts (data not shown); likely a result of their acclimation capacity associated with root elongation, maturation, and water uptake capacity (Venema et al., 2008; Koevoets et al., 2016). Stomatal response to cold soils was regarded as an important mechanism for chill tolerance (Bloom et al., 2004), but under longer exposure of roots to cold soils, it appears that a root-to-shoot interplay in water relations from the acclimation period resulted in the observed decreases of *g*_s_ under SST. As roots acclimated to cold soils, *g*_s_ adjusted to the capacity of the roots to provide water (i.e., *K*_R_) for transpiration demands, and this was supported by a decreased discrimination of ^13^C under SST. In addition, the relative contributions of the hydrostatic and osmotic driven flow are important and could fluctuate on a diurnal scale (Knipfer and Fricke, 2011). Early in the day, the lower soil temperatures increase water viscosity and resistance for water movement toward the root (lower *Lp*^∗^_hyd_), which may result in an increased contribution of the cell-to-cell pathway (e.g., increased osmotic adjustment) to support *g*_s_ and *P*_n_. If roots lack the capacity to acclimate (e.g., in SUP), growth rates may be reduced and unutilized carbohydrates can accumulate, resulting in the down regulation of photosynthesis (Equiza et al., 1997; Venema et al., 1999).

Increases in root-to-shoot ratios are a common response to a variety of abiotic stresses such as soil chilling (Venema et al., 2008), drought (Xu et al., 2015), salinity (Maggio et al., 2007), and heat (Equiza et al., 2001), indicating that under abiotic stress more roots are needed to support shoot functions. Our study showed that this was the case with most phenotypes under SST except for the lowest performing phenotype. Increases in root-to-shoot ratio have been shown before for *S. lycopersicum*, although the change was lower compared to wild relatives that originated in colder environments (e.g., *S. habrochaites*; Venema et al., 2008). In our study, the root-to-shoot ratio under SST was driven by larger and more consistent reductions in shoot biomass than decreases in root biomass across phenotypes. The tradeoff of increased root-to-shoot ratios at this early stage of crop establishment may be increased C allocation to support root function at the cost of lower C allocation for shoot growth, which could reduce the total capacity for C assimilation. For cultivated tomato, early canopy growth is associated with increases in yield (Barrios-Masias and Jackson, 2014) as observed in our field data. Thus, crop improvement for chill tolerance should not only consider increases in root biomass (and root-to-shoot ratios), but also on maximizing the functionality and capacity of a smaller root system to provide resources to the shoot.

Shoot N concentration significantly decreased in the lower performing phenotypes (i.e., BHN and SUP), which may have resulted in reduced utilization of photosynthates and a higher accumulation of total non-structural carbohydrates in leaves and stems (Venema et al., 2008; Royer et al., 2013). Reduced leaf N concentrations could also lead to reduced gas exchange as higher N content was correlated with higher rates of *g*_s_ (Spearman correlation coefficient: 0.669). As C demands decreased and starch concentrations increased in leaves, downregulation of photosynthesis (e.g., SUP) can further decrease plant growth and result in higher C-to-N ratios. Although the shoot C concentration under SST was higher for most phenotypes, it was a small increase (<4%) over the OST, but N concentrations decreased at least 14% and primarily explained the observed changes in C-to-N ratios. Generally, SST would result in lower utilization and translocation of nutrients and photosynthates due to decreased root metabolic activity (Hurewitz and Janes, 1983), and reduce the capacity for nutrient and water uptake needed to support early root and shoot growth.

Early season differences in macronutrient content has been related to root growth (Wang et al., 2016), and our results showed that each rootstock had a different effect on the nutrient profile of the common scion. Individual nutrients are known to have specific functions for mitigating temperature-related stress (Waraich et al., 2012). However, we did not investigate any specific nutrient but instead considered whether the nutrient profiles differed and could help explain phenotype performance. This was observed, for instance, in the discriminant analysis (LD1 axis) where one of the best performing phenotypes (MAX), separated from the lower performing ones (e.g., SUP). Plant uptake of nutrients such as P are inadequate under SST likely due to insolubility and a reliance on root surface area (Case et al., 1964; Mackay and Barber, 1984; Cumbus and Nye, 1985), and early P uptake was one of the main nutrients driving differences among phenotypes in the main axis (LD1) of the discriminant analysis. Increased P uptake has been shown to improve photosynthetic parameters under SST (Starck et al., 2000; Zhou et al., 2009). Other nutrients such as Ca may have played a role in the acclimation to chilling temperatures as Ca regulates cell expansion, cell membrane and wall construction, stomatal closure, and activation of ATPase to increase cellular uptake of nutrients (Palta, 1990; Ntatsi et al., 2014). Although early season N concentrations were not obtained, canopy cover was highly correlated with NDVI (Spearman correlation coefficient: 0.958, which can be used as an indicator of plant N content (i.e., chlorophyll) (Ihuoma and Madramootoo, 2019). Grafted phenotypes tended to display lower NDVI a week after transplanting but NDVI of all grafted phenotypes surpassed the non-grafted cultivar within a month indicating that rootstocks increased N uptake and improved plant performance early in the growing season.

Overall, our results indicate that suitable rootstocks can improve plant performance under SST by supporting plant-water relations as well as altering or enhancing nutrient uptake. We showed that root traits associated with water uptake such as higher root biomass and increased *Lp*^∗^_os_ were important to support leaf gas exchange under SST. In addition, changes in the nutrient profile appeared to correlate with improved overall plant performance, which has been demonstrated in other studies (Huang et al., 2016). Although progress is being made on the utilization of tomato wild relatives as a germplasm resource because of their demonstrated performance under cold environments (e.g., *S. habrochaites*) (e.g., Schwarz et al., 2010; Ntatsi et al., 2017), some commercial rootstocks could be used to mitigate the stress of early-season field plantings in cold soils. Further research on the relative importance of root morphology and architecture into the functionality of roots under abiotic stress and the development of genetic markers may help understand rootstock-scion interactions and assist farmers in selecting rootstock and cultivar combinations better suited to their local conditions.

## Data Availability Statement

The raw data supporting the conclusions of this article will be made available by the authors, without undue reservation.

## Author Contributions

SB: investigation, methodology, formal analysis, validation, writing – original draft, review, and editing. LH-E: methodology, writing, review, and editing. M-SB: investigation, writing, review, and editing. FB-M: conceptualization, methodology, resources, supervision, writing, review, and editing. All authors contributed to the article and approved the submitted version.

## Conflict of Interest

The authors declare that the research was conducted in the absence of any commercial or financial relationships that could be construed as a potential conflict of interest.

## Abbreviations

BHN: Non-grafted cultivar BHN-589
DAP: days after planting
EST: Estamino rootstock with BHN-589 scion
MAX: Maxifort rootstock with BHN-589 scion
OST: optimal soil temperature(s)
RST: RST-04-106-T rootstock with BHN-589 scion
SRT: suboptimal root temperature(s)
SST: suboptimal soil temperature(s)
SUP: Supernatural rootstock with BHN-589 scion.
