# Supplementary material for: Tomato Rootstocks Mediate Plant-Water Relations and Leaf Nutrient Profiles of a Common Scion Under Suboptimal Soil Temperatures
Source: Front Plant Sci. 2021 Jan 21;11:618488. doi: 10.3389/fpls.2020.618488 (PMC7859091; doi:10.3389/fpls.2020.618488)

Supplemental Table 1. Standardized discriminant unit coefficients for each linear discriminant (LD1-LD4) from a linear discriminant analysis based on ten nutrients (B, Ca, Cu, Fe, K, Mg, Mn, Na, P, Zn) at 3 times points (26, 62, 126 DAP) and C and N at 126 DAP for four grafted phenotypes (Estamino, Maxifort, RST-04-106-T, and Supernatural) and one cultivar (BHN-589) (n=8). Elements for each linear discriminant are sorted based on feature importance (i.e. magnitude) of their coefficient and total variation explain by each linear discriminant is provided at the top.


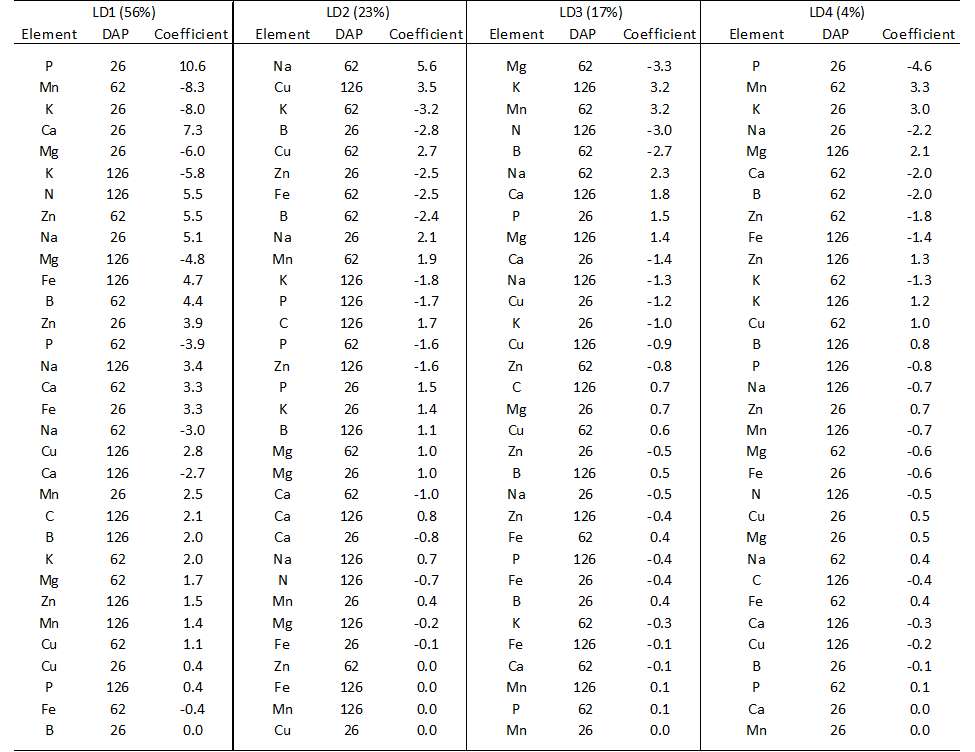

Supplement: Supplementary file 1 [file Table_1.DOCX]
