# Supplementary material for: Tomato Rootstocks Mediate Plant-Water Relations and Leaf Nutrient Profiles of a Common Scion Under Suboptimal Soil Temperatures
Source: Front Plant Sci. 2021 Jan 21;11:618488. doi: 10.3389/fpls.2020.618488 (PMC7859091; doi:10.3389/fpls.2020.618488)
Supplement: Supplementary file 2 [file Table_2.DOCX]

Supplemental Table 2. Mean nutrient concentration of tomato leaf samples at 26, 62, and 126 DAP for four grafted phenotypes (Estamino, Maxifort, RST-04-106-T, and Supernatural) and one cultivar (BHN-589) (n=8) used to conduct a linear discriminant analysis.
